# Genetic characterization of fluoroquinolone-resistant *Escherichia coli* associated with bovine mastitis in India

**DOI:** 10.14202/vetworld.2016.705-709

**Published:** 2016-07-11

**Authors:** Sangeetha Balakrishnan, Prabhakar Xavier Antony, Hirak Kumar Mukhopadhyay, Raghavan Madhusoodanan Pillai, Jacob Thanislass, Vijayalakshmi Padmanaban, Mouttou Vivek Srinivas

**Affiliations:** 1 Department of Veterinary Microbiology, Rajiv Gandhi Institute of Veterinary Education and Research, Puducherry - 605 009, India; 2 Department of Veterinary Biochemistry, Rajiv Gandhi Institute of Veterinary Education and Research, Puducherry - 605 009, India; 3 Department of Veterinary Medicine, Rajiv Gandhi Institute of Veterinary Education and Research, Puducherry - 605 009, India

**Keywords:** *Escherichia coli*, fluoroquinolones, *gyrA*, *parC*, quinolone resistance determining region

## Abstract

**Aim:**

The present study was undertaken to characterize the mutation in *gyrA* (DNA gyrase) and *parC* (topoisomerase IV) genes responsible for fluoroquinolone resistance in *Escherichia coli* isolates associated with the bovine mastitis.

**Materials and Methods:**

A total of 92 milk samples from bovine mastitis cases were sampled in and around Puducherry (Southern India). Among these samples, 30 isolates were bacteriologically characterized as *E. coli*. Minimum inhibitory concentrations (MIC) of fluoroquinolones of these 30 *E. coli* isolates were evaluated by resazurin microtiter assay. Then, the quinolone resistance determining region (QRDR) (*gyrA* and *parC* genes) of these *E. coli* isolates was genetically analyzed for determining the chromosomal mutation causing fluoroquinolone resistance.

**Results:**

*E. coli* isolates showed a resistance rate of 63.33%, 23.33% and 30.03% to nalidixic acid, ciprofloxacin and enrofloxacin, respectively. Mutations were found at 83^rd^ and 87^th^ amino acid position of *gyrA* gene, and at 80^th^ and 108^th^ amino acid position of *parC* gene in our study isolates. Among these five isolates, one had a single mutation at 83 amino acid position of *gyrA* with reduced susceptibility (0.5 µg/ml) to ciprofloxacin. Then, in remaining four isolates, three isolates showed triple mutation (at *gyrA*: S83⟶L and D87⟶N; at *parC*: S80⟶I) and the fifth isolate showed an additional mutation at codon 108 of *parC* (A108⟶T) with the increased ciprofloxacin MIC of 16-128 µg/ml. The most common mutation noticed were at S83⟶L and D87⟶N of *gyrA* and S80⟶I of *ParC*.

**Conclusion:**

The study confirms the presence of mutation/s responsible for fluoroquinolone resistance in QRDR of *gyrA* and *parC* genes of *E. coli* isolates of animal origin, and there is increased rate of fluoroquinolone resistance with high-level of MIC. The mutations observed in this study were similar to that of human isolates.

## Introduction

Quinolones and fluoroquinolones are commonly used antimicrobial agent in both human and veterinary medicine due to its excellent safety profile and their broad spectrum activity against Gram-negative, Gram-positive, mycobacterial pathogen as well as anaerobes [1]. Resistance to quinolones is mainly due to a chromosomal mutation in DNA gyrase of *gyrA* and topoisomerase IV of *parC* gene, even though other mechanisms such as efflux pump and plasmid-mediated resistance are reported [2-5]. These chromosomal mutations are mostly noticed in the highly conserved domain of N-terminus region of *gyrA* and its analogs region of *parC* gene, known as quinolone resistance determining region (QRDR) [5-7].

Before the early 1990s, fluoroquinolone resistances are rarely noticed, but successful treatment outcomes resulted in an overuse of fluoroquinolones, led to an increased rate of resistance [8]. In countries like India, antimicrobial agents are widely used in food animals as a growth promoter. This non-therapeutic use of antibiotics especially fluoroquinolones are common and as such there is no proper regulation regarding the use of antibiotics in livestock industry [9]. Nowadays, the fluoroquinolone resistance has been reported worldwide [3,10,11].

Hence, the present study was aimed at investigating the occurrence of fluoroquinolone resistance associated with bovine coliform mastitis in Puducherry (Southern India) and detecting the genetic mutations as well as correlations responsible for their resistance from *Escherichia coli* isolates of animal origin.

## Materials and Methods

### Ethical approval

The approval from the Institutional Animal Ethics Committee (IAEC) to carry out this study was not required as no invasive technique was used. Milk samples were being collected from mastitis affected bovines from Veterinary hospitals and Veterinary Dispensaries in Puducherry.

### Isolation and identification of E. coli

A total of 92 milk samples were collected from mastitis affected bovines in Puducherry (Southern India) aseptically in sterile vials and cultured in MacConkey’s agar/Eosin methylene blue agar for isolation of the organism. The isolated Gram-negative bacteria were identified up to genus level based on the morphology, cultural and biochemical reactions. All the isolates were identified up to species level based on the standard bacteriological methods described by Barrow and Feltham [11] and were further confirmed by polymerase chain reaction (PCR) employing species-specific primer targeting the alanine racemase gene (*alr* gene) of *E. coli* [12] with the amplicon size of 366 bp (Table-1). Isolates which were positive by species-specific PCR analysis were stored in glycerol Luria broth at −40°C for further analysis.

**Table-1:** Oligonucleotide primer sequence used for amplification of the following target gene in this study.

Primers	Primer sequence	Target gene	Size (bp)	Reference
Alr-F	5’CTGGAAGAGGCTAGCCTGGACGAG3’	alr gene	366 bp	[12]
Alr-R	5’AAAATCGGCACCGGTGGAGCGATC3’			
GyrA-F	5’ACGTACTAGGCAATGACTGG3’	gyrA gene	190 bp	[16]
GyrA-R	5’AGAAGTCGCCGTCGATAGAAC3’			
ParC-F	5’TGTATGCGATGTCTGAACTG3’	parC gene	265 bp	[16]
ParC-R	5’CTCAATAGCAGCTCGGAATA3’			

### Fluoroquinolone susceptibility testing

The minimum inhibitory concentration (MIC) of nalidixic acid, ciprofloxacin, and enrofloxacin were evaluated for the 30 *E. coli* isolates by resazurin microtiter assay, which is an indicator based broth microdilution method [13]. The test uses resazurin indicator, cation adjusted Mueller-Hinton broth, bacterial suspension with the concentration of 5 × 10^7^ cfu/ml and *E. coli* ATCC 25922 as a control strain. The results were interpreted according to the performance and interpretative guidelines of the Clinical and Laboratory Standard institute [14].

### Genetic amplification of gyrA and parC genes

Five isolates with different ciprofloxacin MIC ranging from 0.5 to 128 µg/ml were selected for genetic analysis of fluoroquinolone resistance. For genetic analysis, the QRDR of the *gyrA* gene and analogs *parC* genes was amplified (primers listed in Table-1) with the amplicon size of 190 bp and 265 bp, respectively [15,16]. The template DNA from the *E. coli* isolates was extracted by boiling lysis method for the PCR assay [17]. The PCR-amplification of QRDR regions was carried out using 100 ng template DNA, 5 µl ×10 PCR buffer, 2 mM MgCl_2_, 1 µl of 10 mM deoxynucleotide triphosphates, 10 µM of forward and reverse primers, 2U of Taq DNA Polymerase (New England Bio Labs) and the volume was made up to 50 µl with nuclease free water. The template DNA from *E. coli* ATCC 25922 strain was used as positive control. The PCR amplification consisted of initial denaturation at 95°C for 5 min followed by 35 cycles of denaturation at 95°C for 30 s, annealing at 55°C for 30 s, and extension at 68°C for 1 min, followed by a final extension at 68°C for 10 min. The PCR amplified products were resolved on 1.5% agarose gel in tris acetate ethylenediaminetetraacetic acid buffer and visualized under ultraviolet transilluminator (Syngene, UK).

### Genetic analysis

The amplified PCR products targeting two different genes for the five isolates were gel purified using a by Perfectprep gel cleanup kit (Eppendorf) and custom sequenced for both directions (5’-3’ and 3’-5’) with the same set of the primer pair, using the Automated Sequencer, Applied Biosystem 3100. The specificity of the sequences obtained, the nucleotide variations and amino acid variations with respect to the gyrA and parC genes sequence of *E. coli* strain were determined using basic local alignment search tool (http://blast.ncbi.nlm.nih.gov/Blast.cgi). The obtained nucleotide sequences of gyrA and parC genes of five isolates were aligned with the sequence of *E. coli* K-12 MG1655 genome using ClustalW (www.ebi.ac.uk/clustalw) and analyzed for the nucleotide variation in their gene. The 10 sequences (5 gyrA and 5 parC genes sequence) under the study were submitted in Genbank under the accession numbers JX446586, JX446587, JX446591, JX446592, JX446594 (*gyrA* gene); JX446596, JX446597, JX446601, JX446602, JX446604 (*ParC* gene).

## Results and Discussions

**Figure-1:**
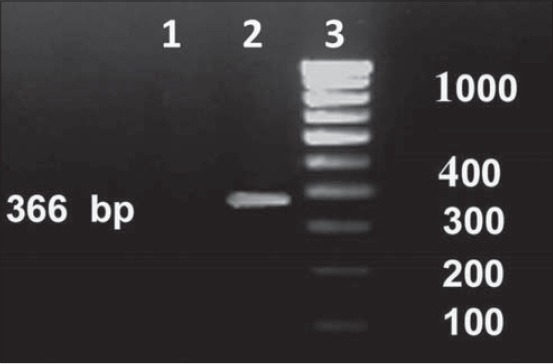
Screening of isolates for detection of *Escherichia coli* using species-specific primers targeting the alanine racemase gene (alr gene). Lane 1: Negative isolate, Lane 2: Positive isolate, Lane 3: DNA ladder.

Out of 92 milk samples collected from mastitis case, 30 were successfully isolated and confirmed to be *E. coli* with the species-specific PCR analysis (Figure-1).

### Fluoroquinolone susceptibility testing

The MIC results for the 30 *E. coli* isolates to nalidixic acid, ciprofloxacin, and enrofloxacin were showed in Table-2. The MIC resistant breakpoint for nalidixic acid, ciprofloxacin, and enrofloxacin was >32, ≥4, and ≥2 µg/ml, respectively. The MIC value for nalidixic acid ranged from 4 to >1024 µg/ml. Of the 30 *E. coli* isolates, 17 (56.67%) isolates required MIC of ≥256 µg/ml and 2 (6.67%) isolates required 64 µg/ml nalidixic acid for inhibition of growth, and remaining 11 isolates showed MIC of 4-16 µg/ml, which is below the resistance breakpoint. The MIC value for ciprofloxacin ranged from 0.0625 to 128 µg/ml. Out of 30 *E. coli* isolates, 7 (23.33%) isolates were resistant to ciprofloxacin, which required 4-128 µg/ml of ciprofloxacin for inhibition of *E. coli*. The MIC value for enrofloxacin ranged from 0.03 to 512 µg/ml. Of the 30 *E. coli* strains, 10 (30.03%) isolates were found to be resistant to enrofloxacin, which required 2-512 µg/ml of enrofloxacin for inhibition. 7 (23.33%) isolates were cross-resistant to ciprofloxacin as well as to enrofloxacin. In 2010, Ranjan *et al*. [18] reported low fluoroquinolone resistance rate with the cure rates of 91.67% for enrofloxacin and 90.15% for ciprofloxacin (90.15%) in the treatment of clinical bovine mastitis, whereas, in our study, 63.33% of the *E. coli* isolates were resistant to nalidixic acid, 23.33% to ciprofloxacin and 30.03% to enrofloxacin, which is in increasing side. It is observed that 19 isolates which were resistant to nalidixic acid (≥32 µg/ml to ≥1024 µg/ml), showed either resistance or reduced susceptibility to fluoroquinolones used in our study. According to Livemore [19], isolates with reduced susceptibility to fluoroquinolones may become resistant in the period of time and evolution of such reduced susceptibility to quinolone is a serious concern due to rapid rising rates of fluoroquinolone resistance in many parts of the world. Resistance to one fluoroquinolone may confer resistance to other fluoroquinolones [20]. In our study, 23.33% of the isolates were cross-resistance to ciprofloxacin as well as enrofloxacin.

**Table-2:** MIC value of *E. coli* isolates from bovine mastitis milk in this study.

Isolate	MIC (µg/ml)		

	Nalidixic acid	Ciprofloxacin	Enrofloxacin
CM1	>1024	128	512
CM2	1024	2	0.125
CM3	8	0.0625	0.0625
CM4	1024	0.5	4
CM5	64	0.25	0.25
CM6	1024	0.5	8
CM7	16	0.5	0.5
M2	16	2	0.03
M3	16	0.25	0.25
SM3	16	0.25	0.25
BM1	16	0.25	0.25
BM7	1024	0.5	0.5
KM1	4	0.06	0.06
TVM8	1024	32	128
AM1	8	0.0625	0.0625
AM2	8	0.0625	0.0625
AM3	256	2	1
AM4	1024	2	2
AM5	1024	0.5	1
AM6	1024	16	32
AM7	>1024	128	256
AM8	1024	2	1
AM9	1024	32	128
AM10	1024	1	0.5
AM11	1024	8	32
AM12	1024	16	64
SMM6	16	0.5	0.5
MM1	4	0.06	0.06
MM5	64	0.25	0.25
MM8	1024	0.5	1

*E. coli*=*Escherichia coli*, MIC=Minimum inhibitory concentrations

### Genetic analysis

**Figure-2:**
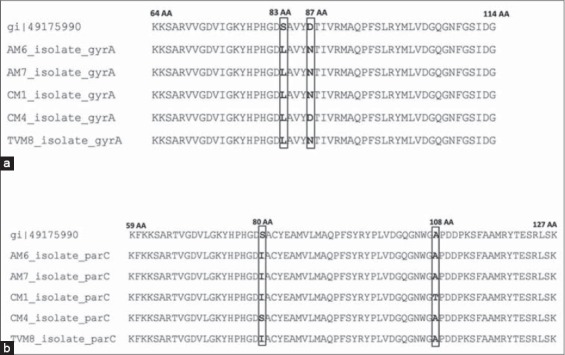
Amino acid variation of (a) *gyrA* gene and (b) *parC* gene of five *Escherichia coli* isolates under this study and wild type *E. coli* reference strain (49175990) available in GenBank.

Sequence analysis of the 190 bp PCR product size covering the QRDR of *gyrA* showed the presence of mutation at codon 83 Ser⟶Leu (TCG⟶TTG) of *gyrA* gene in all five isolates sequenced (Figure-2a). Several studies reports that the substitution at codon 83 is the most common and consistent mutation found in the fluoroquinolone-resistant *E. coli* strains [3,17,20]. Four out of five isolates (except CM4) also had a second mutation at 87^th^ codon Asp⟶Asn of *gyrA* gene (GAC⟶AAC). Mutation at these two codons (83^rd^ and 87^th^ codon) of GyrA protein is responsible for causing high-level of quinolone resistance than the mutation at other codons within the QRDR [20].

With respect to *parC*, isolate CM4, which had a single mutation in *gyrA* did not have any mutation in QRDR of *parC* gene, whereas the rest four isolates having double mutation in *gyrA* gene also had mutation/s in *parC* gene (Figure-2b). Among five isolates, four had a third mutation at codon 80 Ser⟶Ile (AGC⟶ATC) of QRDR of *parC* gene, which is analogous to 87^th^ codon of *gyrA* gene. Out of four isolates bearing a third mutation in *parC*, one isolates (CM1) had a fourth mutation at 108^th^ codon Ala⟶Thr (GCG⟶ACG). One isolates (CM4) with a single mutation in *gyrA* did not exhibit any mutation in *parC*. This is because according to the currently accepted alternating-target model, high-level fluoroquinolone resistance in *E. coli* develops by stepwise acquisition of target mutations, in which DNA gyrase is the primary target and topoisomerase IV is the secondary target for fluoroquinolones in *E. coli* [21-23]. Most common mutation noticed were at S83⟶L and D87⟶N of *gyrA* and S80⟶I of *ParC* in our study (Table-3).

**Table-3:** MIC values and amino acid variations at QRDR (*gyrA* and *parC* gene) of fluoroquinolone-resistant *E. coli* isolates.

Isolate	MIC (µg/ml)			Amino acid variation			

				*gyrA* gene		*parC* gene	
		
	NAL	CIP	ENR	83	87	80	108
Reference strain (49175990^a^)				S	D	S	A
CM4	1024	0.5	4	L	D	S	A
AM6	1024	16	32	L	N	I	A
TVM8	1024	32	128	L	N	I	A
AM7	>1024	128	256	L	N	I	A
CM1	>1024	128	512	L	N	I	T

a Reference strain GenBank accession ID, NAL=Nalidixic acid, CIP=Ciprofloxacin, ENR=Enrofloxacin, S=Serine, L=leucine, D=Aspartic acid, N=Asparagine, I=Isoleucine, A=Alanine, T=Threonine, QRDR=Quinolone resistance determining region, *E. coli=Escherichia coli*, MIC=Minimum inhibitory concentrations

The relationship between the number of mutation/s and their MIC values are shown in Table-3. The correlation between MIC value and mutation pattern were investigated to determine the potential mechanism of fluoroquinolone resistance. The previous studies by different researchers [23] suggest that there is correlation between the number of changes in *gyrA* and *parC* genes and the level of quinolone resistance in *E. coli* strains. Although our study is small to explain the correlation, it is noticed that isolate with single mutation in *gyrA* and no mutation in *parC* showed low-level of fluoroquinolone resistance with ciprofloxacin MIC of 0.5 µg/ml and enrofloxacin MIC of 4 µg/ml, whereas isolates with three mutations (two in *gyrA* and one in *parC*) were associated with moderate to high level of resistance to fluoroquinolone with MIC of ciprofloxacin and enrofloxacin ranging from 16 to 128 µg/ml and 32-256 µg/ml, respectively. One isolate with four mutations (two in *gyrA* and two in *parC*) showed the highest level of resistance to fluoroquinolone with ciprofloxacin and enrofloxacin MIC of 128 and 512 µg/ml; respectively. The amino acid substitutions observed, in this study, were similar to that of mutations reported from isolates of human origin [24].

## Conclusion

Antimicrobial agents have been used in livestock and poultry since the early 1950s to treat infection, improve growth and feed efficiency [18]. Overuse and misuse of antimicrobial agent led to an increased antimicrobial resistance around the world leading to treatment failures in infectious diseases of human and animal. Mutations in QRDR of *gyrA* and *parC* genes are important for developing high-level of fluoroquinolone resistance in *E. coli* isolates. The present study revealed the prevalence of fluoroquinolone resistance in *E. coli* of animal origin. There is increased rate of fluoroquinolone resistance with high-level of MIC and presence of chromosomal mutation/s at QRDR (*gyrA* and *parC* genes) of fluoroquinolone-resistant *E. coli* isolates of bovine mastitis milk samples. These mutation patterns from the animal isolate were similar to that of human isolate with no significant differences.

## Authors’ Contributions

SB and PXA were involved in the design of this research work. The research was done by SB. PXA has monitored all the activities being a supervisor. HKM, RMP, JT, and VP have assisted this research work. SB and MVS drafted and revised the manuscript. All authors read and approved the final manuscript.
